# Impact of Voluntary Folic Acid Fortification of Corn Masa Flour on RBC Folate Concentrations in the U.S. (NHANES 2011–2018)

**DOI:** 10.3390/nu13041325

**Published:** 2021-04-16

**Authors:** Arick Wang, Charles E. Rose, Yan Ping Qi, Jennifer L. Williams, Christine M. Pfeiffer, Krista S. Crider

**Affiliations:** 1 Centers for Disease Control and Prevention, National Center on Birth Defects and Developmental Disabilities, Atlanta, GA 30341, USA; cvr7@cdc.gov (C.E.R.); irv7@cdc.gov (Y.P.Q.); znv8@cdc.gov (J.L.W.); kvc3@cdc.gov (K.S.C.); 2 Oak Ridge Institute for Science and Education, Oak Ridge, TN 37831, USA; 3 Centers for Disease Control and Prevention, National Center for Environmental Health, Atlanta, GA 30341, USA; cfp8@cdc.gov

**Keywords:** corn masa, food fortification, NTDs, folic acid

## Abstract

Surveillance data have highlighted continued disparities in neural tube defects (NTDs) by race-ethnicity in the United States. Starting in 2016, the Food and Drug Administration (FDA) authorized voluntary folic acid fortification of corn masa flour to reduce the risk of neural tube defects (NTDs) among infants of Hispanic women of reproductive age. To assess the impact of voluntary corn masa fortification, cross-sectional data from the National Health and Nutrition Examination Survey (NHANES) 2011–2018 for Hispanic women of reproductive age with available red blood cell (RBC) folate concentrations were analyzed, with additional analyses conducted among Hispanic women whose sole source of folic acid intake was fortified foods (enriched cereal grain products (ECGP) only), excluding ready-to-eat cereals and supplements. RBC folate concentration (adjusted geometric mean) among Hispanic women of reproductive age did not differ between 2011–2016 and 2017–2018, though RBC folate concentration increased significantly among lesser acculturated Hispanic women consuming ECGP only. Concentrations of RBC folate for those born outside the U.S and residing in the U.S <15 years increased from 894 nmol/L (95% CI: 844–946) in 2011–2016 to 1018 nmol/L (95% CI: 982–1162; *p* < 0.001) in 2017–2018. Primarily Spanish-speaking Hispanic women of reproductive age who only consumed ECGP saw an increase from 941 nmol/L (95% CI: 895–990) in 2011–2016 to 1034 nmol/L (95% CI: 966–1107; *p* = 0.03) in 2017–2018. By subpopulation, we observed no significant changes in the proportion at risk of NTDs (<748 nmol/L) and no changes in the model-based estimated NTD rates following voluntary corn masa fortification. This analysis suggests that there is a remaining risk among Hispanics for folate sensitive NTDs, though continued monitoring of folate status in future NHANES data cycles will help inform the long-term efficacy of voluntary fortification of corn masa flour.

## 1. Introduction

Folic acid consumed prior to and during the first months of pregnancy has been shown to reduce the risk of serious birth defects of the brain and spinal cord, collectively referred to as neural tube defects (NTDs) [1,2]. As a result, the U.S. Centers for Disease Control and Prevention (CDC) and the World Health Organization (WHO) recommend that women capable of becoming pregnant consume ≥400 μg/d synthetic folic acid, in addition to foods rich in natural folates, to prevent NTDs [3].

With negligible costs and no needed behavioral change or required access to health care, food fortification is an established means of reducing health disparities associated with micronutrient deficiencies. In 1996, the Food and Drug Administration (FDA) authorized the mandatory folic acid fortification of grain products labeled as enriched, requiring 140 μg folic acids/100 g of product [4]. Fortification of this staple food was implemented in the U.S. because about 45% of pregnancies in the U.S. are unplanned, and the neural tube closes within the first month of pregnancy before many women know they are pregnant [5,6]. Data from birth defects surveillance systems have shown that, since the implementation of mandatory fortification of enriched grain products, spina bifida and anencephaly prevalence has decreased from 10.7 to 5/10,000 live births, averting >1300 cases/year and saving an estimated >$600 million/year [7,8,9].

Despite reductions in disparities since the fortification of enriched grain products, disparities have persisted. Hispanic women still have the highest NTD prevalence compared to other racial/ethnic groups [10]. Post-fortification studies have found that Hispanic women are less likely to consume folic acid from fortified foods, as fortification does not include corn masa flours, which are a main staple among Hispanic populations in the U.S. [11,12]. Studies have also shown that acculturation, an individual’s experience entering a new culture, is a mediating factor for NTD risk among infants of Mexican-American women, the largest subgroup of Hispanics in the U.S. [12]. In 2016, the March of Dimes, in collaboration with corn masa manufacturer GRUMA, successfully petitioned the FDA to allow for the voluntary fortification of corn masa flours with folic acid in order to reduce NTD-affected pregnancies among lesser acculturated Mexican-American women [13].

Red blood cell (RBC) folate concentrations are associated with NTD risk. NTD risk decreases by up to 10-fold as RBC folate concentrations move from deficiency into optimal concentrations [9]. In 2015, the WHO established the RBC folate concentration of 906 nmol/L as the threshold for optimal NTD prevention in populations. This threshold can be used to monitor the need for and effectiveness of folic acid fortification programs [14]. We evaluated the impact of voluntary corn masa fortification on RBC folate concentrations and predicted NTD risk among Hispanic women of reproductive age in the pre- (National Health and Nutrition Examination Survey (NHANES) 2011–2016) and post- (NHANES 2017–2018) voluntary fortification periods in the U.S. Additionally, we looked at differences among Hispanic women of reproductive age whose sole folic acid source was fortified foods, the target subpopulation for the intervention, to determine if voluntary fortification of corn masa flour significantly impacted RBC folate concentrations and NTD risk.

## 2. Materials and Methods

### 2.1. Demographic Characteristics and RBC Folate Concentrations

Cross-sectional NHANES data from 2011 to 2018 were collected using a stratified multistage probabilistic design, and data collection in 2-year cycles (2011–2012, 2013–2014, 2015–2016, 2017–2018) was used. The pre-fortification time period (NHANES 2011–2016) was determined to be a stable time period to provide a baseline [15]. Each 2-year NHANES cycle captured a nationally representative sample of the civilian U.S. population. Respondents participated in household interviews and in person physical examinations at a mobile examination center (MEC). Response rates among women of reproductive age (WRA) ranged from 47.1% to 77.1% depending on the survey cycle and age group; detailed interview and examination response rates are publicly available [16]. Detailed descriptions of the survey have been published elsewhere [17,18,19,20,21,22,23].

RBC folate concentrations were measured using a microbiologic assay [17]; concentrations were log-transformed for the analysis. Similar to previous analyses, RBC folate concentrations were categorized into risk of NTDs: (1) high risk, ≤585 nmol/L in the CDC NHANES assay, associated with >14 NTDs/10,000 live births; (2) elevated risk, 586–747 nmol/L, associated with 9–14 NTDs/10,000 live births; (3) optimal, 748–1215 nmol/L, associated with <9 NTDs/10,000 live births; and (4) limited additional benefits, ≥1216 nmol/L. Additionally, RBC folate concentration was analyzed as a dichotomous variable by NTD risk, with high and elevated NTD risk having RBC folate concentrations of <748 nmol/L, and low NTD risk having RBC folate concentrations of ≥748 nmol/L [24]. This dichotomized value was determined using the NHANES assay, which is equivalent to dichotomizing at 906 nmol/L on the Molloy assay [9].

Information on age, body mass index (BMI), race/ethnicity, education level, poverty-income ratio (PIR), language preference, country of origin, and length of time residing in the U.S. was obtained through a household interview questionnaire. Education was collapsed into three categories: having less than a high school education, having a high school diploma or GED with some or no college, and having at least a college degree. PIR was based on family reported household income and household size; ratios of <1.0 were considered to be below the official poverty threshold. Hispanic participants could identify as either Mexican Americans or other Hispanics, providing two ethnic groups within the Hispanic subpopulation.

For the purpose of this analysis, data from 2011–2018 were combined and MEC weights were combined into an 8-year weight. Data were then stratified into pre- (2011–2016) and post- (2017–2018) voluntary fortification periods. The subpopulation for this analysis consisted of nonpregnant women aged 12–49 years who were seen at the MEC, and for whom RBC folate data were available (see Supplementary Figure S1). Adjusted geometric means (aGM) of RBC folate concentrations were modeled using proper combined MEC survey weights; aGM were separately modeled for all women of reproductive age, Hispanic women of reproductive age, and Hispanic women of reproductive age who consumed only ECGP. All participants in NHANES provided written informed consent.

### 2.2. Acculturation Factors

Among individuals who identify themselves as Hispanic, NHANES includes data collected via telephone interview on the primary language(s) spoken at home, the country of origin, and, among those who identified a country of origin other than the U.S., the length of time they have resided in the U.S was also included. Language at home was categorized into three levels: (1) women who reported speaking English at home all or most of the time, (2) women who reported speaking an equal amount of English and Spanish at home, and (3) women who reported speaking Spanish at home all or most of the time. Women who reported speaking English at home were used as the reference group for calculating adjusted odds ratios.

Country of origin indicated whether an individual was born within or outside the U.S. For women born outside the U.S., data on how long women had been living in the U.S. were available. Like previous studies, time residing in the U.S. was dichotomized as residing in the U.S. <15 years or residing in the U.S. ≥15 years. In order to capture all Hispanic women, a derived categorical variable with three mutually exclusive levels was created: (1) women born within the U.S., (2) women born outside the U.S. and residing in the U.S. ≥15 years, and (3) women born outside the U.S. and residing in the U.S. <15 years. Women born within the U.S were used as the reference group for calculating adjusted odds ratios.

### 2.3. Dietary Factors

Dietary information was available from two 24-h dietary recalls in which individual foods, nutritional information, and food components consumed were recorded. The first 24-h dietary recall was conducted in the MEC, and the second was conducted via telephone interviews 3–4 days afterwards [18,19,20,21,22,23]. As in previous studies, USDA food codes and recorded folic acid consumption were used to categorize primary folic acid intake into four levels: (1) women whose sole source of dietary folic acid was from background enriched cereal grain product fortification (ECGP only); (2) women who took a supplement in addition to background fortification (ECGP + SUPP); (3) women who ate ready-to-eat (RTE) cereals in addition to background fortification (ECGP + RTE); and finally, (4) women who received folic acid from background fortification, RTE cereals, and supplementation (ECGP + SUPP + RTE). For adjusted odds ratios, “ECGP only” was used as a reference group.

Corn masa consumption status was determined using the individual food codes from the 24-h dietary recalls and included cooked corn masa flour and foods that contained corn tortillas and/or corn masa dough. Corn masa consumption was further separated by folic acid supplementation for four distinct levels: (1) women who did not consume either corn masa or folic acid supplements (No masa), (2) women who did not consume corn masa but consumed folic acid supplements (No masa + SUPP), (3) women who consumed corn masa with no additional supplements (Masa), and (4) women who consumed both corn masa and supplements (Masa + SUPP). For calculating adjusted means, “No masa” was used as the reference group.

### 2.4. Usual Intake

Usual intakes for total folic acid consumption were estimated using the National Cancer Institute (NCI) method utilizing the two 24-h dietary recalls and 30-day supplement status [25]. A one-part amount model was used to calculate the median and interquartile range of folic acid consumption for groups stratified by pre- and post-voluntary fortification and by either acculturation factors or corn masa consumption. Age, PIR, BMI, education, and race/ethnicity were used as covariates for the NCI model, accounting for weekday/weekend consumption.

### 2.5. Statistical Analysis

Statistical analyses were conducted using R, version 3.6.1 (Vienna, Austria) [26], in conjunction with the survey package, version 4.0 [27]. Analyses of demographic information, acculturation factors, and RBC folate distributions used corresponding NHANES sample weights to account for probability selection and nonparticipation. An 8-year combined MEC weight was calculated from NHANES, which provided 2-year MEC weights, and was used to calculate all geometric means, frequencies, Chi-squared tests, and Student t-tests. Logistic regression models were used to compute adjusted odds ratios (aOR) and 95% confidence intervals (CI). Adjustments were made to account for differences in education, ethnicity, age, PIR, and BMI. Quantitative variables (age, PIR, BMI) were analyzed continuously. Missing data were excluded from analyses and no formal adjustments were made for multiple comparisons.

Predicted NTD prevalence based on RBC folate concentrations was estimated using the distributions of RBC folate among Hispanic women of reproductive age overall and among the most at-risk of the subgroups, as previously described, and was adjusted with the 8-year combined MEC weights for NHANES 2011–2018 [28]. In brief, NTD prevalence was predicted within each subgroup using a two-step process. First, hypothetical RBC folate concentrations for 100,000 women were simulated to match the distribution calculated for the subgroup. Second, the simulated RBC folate concentrations were used to estimate NTD prevalence using a Bayesian approach developed from a previously analyzed large prospective dataset [9]. These simulations were conducted using SAS 9.4.

## 3. Results

There were 2070 Hispanic women of reproductive age (12–49 years) in NHANES for whom folate concentrations were available, with 1643 Hispanic women in NHANES 2011–2016, before voluntary corn masa flour fortification, and 427 in NHANES 2017–2018, after the implementation of voluntary corn masa flour fortification (Table 1). The groups across the two periods were comparable by age, BMI, smoking, folic acid supplement use, and primary language spoken at home. In general, participants in NHANES 2017–2018 were more educated, less impoverished, relied more on background enriched grain product fortification for folic acid, and had been in the U.S. longer (among non-U.S. born participants) than NHANES 2011–2016 participants (*p* < 0.05). Overall, there were no changes in the proportion of Hispanic women in each RBC folate risk category (Table 1).

Usual intake of folic acid was modeled and stratified by masa consumption and acculturation factors among Hispanic women of reproductive age, adjusted for age, BMI, PIR, education, and ethnicity (i.e., Mexican Hispanic or other Hispanic). There were no significant differences in estimated folic acid intake among Hispanic women of reproductive age overall, nor were there differences within those who had masa flour products in reported dietary recall following the fortification of corn masa products. There were also no differences in estimated folic acid intake among Hispanic women of reproductive age stratified by acculturation factors following the voluntary fortification of corn masa products (Table 2).

Adjustments were made for age, education, PIR, ethnicity, and BMI when examining differences in RBC folate among racial/ethnic groups among all women of reproductive age. Adjusted geometric means (aGM) and 95% CI were calculated for RBC folate concentrations, and stratified by race/ethnicity (Table 3). Weighted percentages for being at risk of NTD (RBC folate concentration <748 nmol/L) and aOR were computed by race/ethnicity, using non-Hispanic white women as the reference group (Table 3). Between NHANES 2011–2016 and NHANES 2017–2018, there were no changes in either the aGM of RBC folate by racial/ethnic group nor in the proportion of the population at risk of NTDs in either racial/ethnic group (Table 3). These analyses were additionally run excluding PIR to assess the potential impact of missing PIR data, though the exclusion of PIR did not significantly alter the outcome of the analyses.

In examining the differences among Hispanic women of reproductive age, the aGM was adjusted for age, education, PIR, ethnicity, and BMI for each stratified subpopulation. We examined the data stratified by demographic, dietary, and acculturation variables. Overall, there were no significant differences noted in the aGM among Hispanic women from NHANES 2011–2016 to NHANES 2017–2018 (Table 4).

Further analyses were conducted for Hispanic women of reproductive age whose folic acid source was primarily through the fortification of staple foods (ECGP only). Adjustments were made for age, education, PIR, BMI, and ethnicity. There was very little overall change in the aGM of RBC folate among Hispanic women of reproductive age only consuming ECGP from NHANES 2011–2016 (938; 95% CI: 916–960) to NHANES 2017–2018 (944; 95% CI: 891–1000; *p =* 0.85; Table 4). When stratifying the subpopulation by the country of origin combined with the length of time residing in the U.S as a proxy acculturation factor, a modest increase was found in RBC folate among non-U.S. born Hispanic women of reproductive age. RBC folate increased from 975 nmol/L (95% CI: 916–1037 nmol/L) in NHANES 2011–2016 to 1039 nmol/L (95% CI: 929–1162 nmol/L) in NHANES 2017–2018 among non-U.S born Hispanic women of reproductive age having resided within the U.S for ≥15 years (*p* = 0.33). A more substantial increase from 894 nmol/L (95% CI: 844–946) in 2011–2016 to 1018 nmol/L (95% CI: 982–1057) in 2017–2018 was observed among non-U.S. born Hispanic women of reproductive age having resided within the U.S. <15 years (*p* < 0.001; Table 5). These changes in the aGM of RBC folate concentrations were associated with modest decreases in the proportion of the subpopulation at risk of NTDs. The proportion of Hispanic women of reproductive age consuming ECGP only, living in the U.S. ≥15 years, and at risk of NTDs decreased from 20.3% (95% CI: 13.6–27.0) in 2011–2016 to 14.8% (95% CI: 5.4–24.2; *p* = 0.35) in 2017–2018, and the proportion of non-U.S born Hispanic women of reproductive age residing in the U.S. <15 years decreased from 27.7% (95% CI: 19.9–35.5) in 2011–2016 to 22.8% (95% CI: 13.7–31.9; *p =* 0.42) in 2017–2018. The aOR of having an RBC folate concentration <748 nmol/L (i.e., high or elevated NTD risk; calculated using the U.S born Hispanic women of reproductive age consuming only ECGP as the reference group) remained unchanged from 2011–2016 to 2017–2018, and all confidence intervals included 1.00 (Table 5).

When examining the role of acculturation using the primary language spoken at home as a proxy, there was a substantial increase in RBC folate among ECGP only consuming Hispanic women of reproductive age who primarily spoke Spanish at home from 941 nmol/L (95% CI: 895–990 nmol/L) in NHANES 2011–2016 to 1034 nmol/L (95% CI: 966–1107 nmol/L) in NHANES 2017–2018 (*p* = 0.03; Table 5). This increase in the aGM of RBC folate was associated with a modest decrease in the percentage of the subpopulation at risk of NTD from 22.6% (95%CI: 17.6–27.6) in 2011–2016 to 15.8% (95% CI: 10.0–21.5) in 2017–2018 (*p* = 0.08; Table 5). The aOR of having an RBC folate concentration <748 nmol/L, with Hispanic women of reproductive age consuming ECGP only, who primarily spoke English at home as the reference group, contained 1 within the 95% CI both in NHANES 2011–2016 and NHANES 2017–2018 (Table 5). When stratified by masa consumption, there were no changes in RBC folate or NTD risk pre- versus post-voluntary fortification periods (Table 5).

Additional model-based Bayesian estimations of the NTD rate from RBC folate distributions were simulated to provide an estimated rate of NTDs/10,000 live births. Overall, after post-voluntary fortification (NHANES 2017–2018), the estimated NTD rate was 6.3 NTDs/10,000 (95% uncertainty interval (UI): 4.6–8.1) among non-Hispanic white women of reproductive age compared to 6.9 NTDs/10,000 (95% UI: 5.1–8.9) among Hispanic women of reproductive age overall. When limiting to WRA who relied solely on ECGP as a folic acid source, the estimated NTD rate was 7.1 NTDs/10,000 (95% UI: 5.3–9.2) among non-Hispanic white women of reproductive age compared to 7.5 NTDs/10,000 (95% UI: 5.6–9.6; *p =* 0.37) among Hispanic women of reproductive age in the post-fortification time period (Table 6).

## 4. Discussion

The current analyses explore the impact of voluntary corn masa fortification, with data from a single NHANES survey cycle available post-fortification. We found no substantial increases in RBC folate concentrations or substantial reductions in NTD risk or predicted prevalence among Hispanic women of reproductive age after the implementation of the voluntary folic acid fortification of corn masa flour. There were also no substantial changes in the estimated usual intake of folic acid among Hispanic women of reproductive age overall or among those consuming ECGP only who reported consuming corn masa.

However, there were moderate increases in RBC folate among lesser acculturated Hispanic women of reproductive age (i.e., Hispanic women who primarily speak Spanish at home or who have resided in the U.S <15 years) whose sole folic acid intake source was fortified foods, and who were the targeted subpopulation for this fortification intervention. These modest changes did not substantially impact the proportion of the population with folate insufficiency. Estimated NTD rates did not differ between pre- and post-voluntary fortification time periods among Hispanic women of reproductive age whose sole folic acid source was fortified foods. Masa consumption in this target population also showed no substantial change in estimated NTD rates. We were unable to estimate NTD rates among lesser acculturated Hispanic women of reproductive age consuming ECGP only due to sample size limitations in the post-voluntary corn masa flour fortification time period. Overall, estimated NTD rates also remained slightly higher than those of non-Hispanic white women of reproductive age within the post-voluntary fortification time period. While these model-based point estimations were not intended to detect very small differences at the lower end of NTD rates, they indicate that, at least in this initial state, the voluntary fortification of corn masa flour is not providing the overall level of NTD prevention as initially intended. The inclusion of more data as they become available in successive NHANES cycles will provide a more complete assessment as to the efficacy of voluntary fortification of corn masa flour.

The implementation of voluntary corn masa flour fortification aimed to provide a regular folic acid source to households relying on corn masa flour as a primary food staple. As previous studies have noted, food acculturation drives dietary and micronutrient differences among Hispanic populations [29,30]. We were limited to the use of proxy measures for acculturation available from NHANES (i.e., primary language spoken at home, length of time in the U.S). Our results demonstrate a limited reduction in NTD risk and predicted prevalence following the voluntary fortification of corn masa flour within the least acculturated subpopulations. Previous studies have reported achievable reduction of folate-sensitive NTDs to a rate of 5 per 10,000 live births, so there may still be additional reductions in preventable folate-sensitive NTDs yet to be achieved (29). With an estimated 886,000 live births among Hispanic women of reproductive age in the U.S. in 2018, a decrease from 6.9 to 5 NTDs per 10,000 live births could roughly prevent an additional 150 folate-sensitive NTDs in the U.S. [31].

The currently available data limit our ability to detect changes in NTD risk and prevalence. Previous models have shown that steady-state RBC folate concentration is not achieved until a median of 9 months after the initiation of folic acid supplementation [32]. Voluntary fortification was permitted to begin in April 2016. However, it is possible that manufacturers chose to fortify later in 2017 or in 2018, or that fortified corn masa products did not have sufficient market penetration until some later date. Given that data are currently available from one 2-year NHANES cycle (2017–2018) following the implementation of voluntary fortification of corn masa flour, it is possible that the sampled population had not achieved a steady-state in RBC folate concentrations, and therefore, changes in NTD risk and predicted prevalence are currently underestimating the true effect. Additionally, because this analysis was based on a single cycle of data post-fortification, the sample size following voluntary fortification may limit the statistical differences observed. Although NHANES 2017-2018 oversampled Hispanic populations, the small sample size necessitated the inclusion of all Hispanic women of reproductive age, with limited information available on ancestral and cultural identity (only Mexican American and other Hispanic were included). The NHANES sampling procedure may not result in a representative sample of the U.S. Hispanic population. Examining women of reproductive age provides a sample of women who may be at risk of NTDs, but the subpopulation is not representative of at-risk pregnancies in the United States. Differences in diet and acculturation experiences among Hispanic subpopulations of differing ancestral and cultural backgrounds could increase the noise within an already small sample, making it more difficult to detect changes in RBC folate concentrations and NTD risk. Finally, we cannot directly attribute increases in RBC folate concentrations to voluntary fortification of folic acid because of the cross-sectional study design of NHANES.

This analysis also cannot determine if the manufacturers are adding folic acid to corn masa products. We found no changes in the estimated usual intake of folic acid among Hispanic women of reproductive age who reported the consumption of corn masa flour products pre- versus post-voluntary fortification. This suggests that any change in the folic acid content among corn masa flour products following voluntary fortification is undetectable in estimated dietary consumption. Nevertheless, the usual intake of folic acid was estimated using USDA food codes and the USDA database, but the provided nutritional information and reference nutritional information for products that contained corn masa flour within this dataset may not accurately reflect the specific nutritional information of individual food items. Additionally, previous research has suggested that very few manufacturers are voluntarily fortifying corn masa flours and corn masa products, which would account for the unchanged usual intake and limited reduction of NTD risk and predicted prevalence we report herein [33,34].

## 5. Conclusions

The fortification of staple grains is an established and successful public health modality to reduce disparities and increase health equality. Low-dose, continuous folic acid fortification of shelf- and heat-stable products prevents folate deficiency anemia that is common in areas and communities without access to fresh produce or stable food stuffs. Additionally, folic acid fortification of enriched grain products has led to a dramatic decrease in NTD prevalence in the U.S. The fortification of alternative food staples, such as corn masa flour, was proposed to target Hispanic women of reproductive age who are at-risk for preventable folate-sensitive NTDs. These analyses indicate that there have been limited changes in NTD risk and predicted prevalence following the implementation of voluntary fortification of corn masa flour in 2017. We showed modest improvements in RBC folate within the least acculturated subpopulation of Hispanic women, though the increases did not translate to significant changes in folate status. Overall, the increases were smaller than one would expect from the addition of 140 mcg/100 g of product; thus, further prevention of folate-sensitive NTDs might be achievable within the Hispanic population.

## Figures and Tables

**Table 1:** Demographic and behavioral characteristics of U.S. Hispanic women of reproductive age (12–49 years), National Health and Nutrition Examination Survey (NHANES) ^a^ 2011–2018, stratified by pre- and post-implementation of corn masa fortification.

		2011–2016		2017–2018	
	*n*	Weighted % (95% CI)	*n*	Weighted % (95% CI)	*p* Value
**Total ^b^**	1643		427		
**Age**					0.40
12–24	770	36.4 (33.6, 39.3)	196	39.7 (34.8, 44.6)	
25–34	329	26.4 (23.8, 29.0)	77	23.4 (18.5, 28.2)	
35–49	544	37.2 (34.7, 39.7)	154	36.9 (32.1, 41.7)	
**Education level**					0.04
<High School	881	46.1 (42.7, 49.5)	213	38.2 (33.6, 42.8)	
High School graduate/GED	281	18.3 (16.0, 20.7)	86	24.6 (18.8, 30.4)	
>High School	477	35.6 (31.9, 39.2)	127	37.2 (30.5, 43.9)	
Missing	4	—	1	—	
**Poverty income ratio**					0.03
<1.0	561	37.2 (33.4, 41.0)	106	27.4 (22.0, 32.8)	
1.0–1.9	400	27.2 (24.2, 30.2)	115	31.3 (21.6, 41.1)	
2.0–3.9	322	23.6 (20.0, 27.3)	72	22.2 (16.0, 28.5)	
≥4.0	142	11.9 (9.1, 14.6)	51	19.1 (15.0, 23.1)	
Missing	218	—	83	—	
**Body mass index (BMI) [kg/m^2^]**					0.915
Underweight (BMI < 18.5)	75	3.4 (2.6, 4.2)	15	3.0 (1.6, 4.4)	
Normal Weight (18.5 ≤ BMI < 25)	524	29.6 (27.2, 32.1)	126	29.7 (26.3, 33.1)	
Overweight (25 ≤ BMI < 30)	441	28.4 (25.8, 31.1)	124	29.6 (24.8, 34.4)	
Obese (BMI ≥ 30)	584	38.5 (35.6, 41.5)	154	37.7 (33.4, 41.9)	
Missing	19	—	8	—	
**Smoking status**					0.93
Not smoking	1503	91.5 (89.5, 93.4)	389	91.3 (87.7, 94.9)	
Smoking	121	8.5 (6.6, 10.5)	33	8.7 (5.1, 12.3)	
Missing	19	—	5	—	
**RBC^b^ folate concentration by risk category**					0.52
High (≤585 nmol/L)	90	5.4 (4.2, 6.6)	25	6.9 (5.5, 8.4)	
Elevated (586–747 nmol/L)	188	11.4 (9.8, 13.0)	49	11.9 (8.0, 15.7)	
Optimal (748–1215 nmol/L)	931	55.7 (53.6, 57.7)	241	53.1 (48.5, 57.7)	
Limited additional benefit (≥1216 nmol/L)	434	27.6 (25.2, 29.9)	112	28.1 (24.2, 32.0)	
**Folic acid (FA) source**					0.016
ECGP only	914	56.0 (53.1, 58.9)	265	63.8 (60.4, 67.3)	
ECGP + SUPP	248	17.0 (15.0, 19.0)	66	17.4 (14.4, 20.3)	
ECGP + RTE	404	22.1 (19.8, 24.5)	80	15.9 (11.4, 20.5)	
ECGP + SUPP + RTE	77	4.9 (3.6, 6.1)	16	2.9 (1.2, 4.5)	
**Corn masa consumption**					0.59
No masa	591	34.9 (31.0, 38.9)	146	38.1 (34.2, 41.9)	
Masa	727	43.2 (39.7, 46.8)	199	41.7 (36.2, 47.1)	
No masa + SUPP	146	9.8 (7.9, 11.7)	34	9.7 (6.8, 12.6)	
Masa + SUPP	179	12.0 (10.1, 13.9)	48	10.6 (8.2, 12.9)	
**FA supplement dose**					0.48
<400	183	12.0 (10.1, 13.9)	53	12.6 (8.6, 16.7)	
≥400	142	9.8 (8.2, 11.4)	29	7.6 (4.5, 10.7)	
None	1318	78.2 (75.7, 80.6)	345	79.8 (76.3, 83.2)	
**Time spent in U.S**					0.018
U.S Born	901	53.0 (48.3, 57.7)	231	58.4 (53.6, 63.1)	
Born outside U.S, been in U.S ≥15 years	293	20.9 (18.6, 23.1)	103	23.9 (19.9, 27.9)	
Born outside U.S, been in U.S <15 years	389	26.2 (22.5, 29.8)	73	17.7 (12.8, 22.7)	
Missing	60	—	20	—	
**Primary language spoken at home**					0.50
Primarily English	655	40.4 (36.8, 44.0)	160	41.4 (35.4, 47.4)	
Equal Spanish and English	337	18.4 (15.9, 21.0)	95	20.6 (16.4, 24.8)	
Primarily Spanish	644	41.1 (37.7, 44.6)	168	38.0 (33.8, 42.2)	
Missing	7	—	4	—	

^a^ Abbreviations: enriched cereal grain product (ECGP), National Health and Nutrition Examination Survey (NHANES), red blood cell (RBC), ready-to-eat cereals (RTE), supplement use (SUPP); ^b^ Excluded those who did not attend the mobile examination center (2011–2016: N = 76, 2017–2018: N = 23) and those who had missing RBC folate concentrations (2011–2016: N = 91, 2017–2018: N = 20); *p*-values determined using Chi-square test.

**Table 2:** Median usual intake (IQR) of folic acid (mcg) among women of reproductive age (12–49 years), NHANES ^a^ 2011–2018.

	2011–2016	2017–2018	*p* Value
**All women of reproductive age**			
Non-Hispanic White	231 (118, 392)	182 (78, 351)	0.90
Hispanic	177 (85, 299)	161 (71, 277)	0.92
**Hispanic women of reproductive age, ECGP-only**			
*Masa consumption*			
No	101 (71, 133)	134 (80, 197)	0.66
Yes	115 (81, 152)	107 (65, 144)	0.86
*Primary Language Spoken at Home*			
Primarily English	121 (91, 154)	121 (83, 162)	0.99
Equal Spanish and English	97 (60, 143)	107 (64, 148)	0.97
Primarily Spanish	100 (68, 132)	114 (31, 172)	0.83
*Time spent in U.S*			
U.S Born	115 (87, 146)	122 (78, 169)	0.93
Born outside U.S, been in U.S ≥15 years	99 (55, 149)	126 (98, 150)	0.87
Born outside U.S, been in U.S <15 years	102 (70, 133)	114 (39, 165)	0.85

^a^ Abbreviations: enriched cereal grain product (ECGP), National Health and Nutrition Examination Survey (NHANES); *p*-values determined using Student’s *t*-test.

**Table 3:** Adjusted red blood cell (RBC) folate concentrations of U.S. women of reproductive age (12–49 years), NHANES ^a^ 2011–2018, stratified by pre- and post-implementation of corn masa fortification.

Race/Ethnicity	Time Period	*n*	Adjusted Geometric Mean ^b^ of RBC Folate, nmol/L (95% CI)	*p* Value (Between Time Periods)	% with RBC Folate Concentration < 748 nmol/L ^c^ (95% CI)	*p* Value (Between Time Periods)	Adjusted Odds Ratio ^d^ of Having an RBC Folate Concentration < 748 nmol/L (95% CI)
Non-Hispanic White	2011–2016	1736	1077 (1044, 1111)		15.2 (12.7, 17.7)		REF
Hispanic		1643	1017 (994, 1040)		16.8 (15.0, 18.5)		1.05 (0.83, 1.33)
Non-Hispanic White	2017–2018	510	1072 (1008, 1141)	0.90	15.2 (9.8, 20.6)	0.99	REF
Hispanic		427	1018 (974, 1063)	0.98	18.8 (14.4, 23.2)	0.39	1.15 (0.45, 2.94)

^a^ Abbreviations: National Health and Nutrition Examination Survey (NHANES), red blood cell (RBC); ^b^ Geometric means adjusted for age, education, poverty-income ratio (PIR), and body mass index (BMI); ^c^ Associated with NTD rate of ≥9 per 10,000 live births; ^d^ Odds ratios adjusted for age, education, PIR, and BMI; *p*-values determined using Student’s *t*-test, examining differences between the two time periods within the race-ethnicity subpopulation.

**Table 4:** Adjusted red blood cell (RBC) folate concentrations of U.S. Hispanic women of reproductive age (12–49 years), NHANES ^a^ 2011–2018, stratified by pre- and post-implementation of corn masa fortification.

		2011–2016		2017–2018	
	*n*	Adjusted Geometric mean ^b^ RBC Folate, nmol/L (95% CI)	*n*	Adjusted Geometric Mean ^b^ RBC Folate, nmol/L (95% CI)	*p* Value
**Total ^c^**	1643	1008 (985, 1031)	427	1006 (963, 1050)	0.93
**Age**					
12–24	770	944 (890, 1000)	196	937 (865, 1015)	0.89
25–34	329	980 (949, 1012)	77	1035 (952, 1126)	0.23
35–49	544	1102 (1030, 1180)	154	1059 (955, 1175)	0.53
**Folic acid source**					
ECGP ^c^ only	914	938 (916, 960)	265	944 (891, 1000)	0.85
ECGP + SUPP	248	1135 (1089, 1182)	66	1147 (1028, 1279)	0.86
ECGP + RTE ^c^	404	1050 (1013, 1090)	80	1038 (993, 1086)	0.70
ECGP + SUPP + RTE	77	1257 (1128, 1401)	16	1352 (1234, 1481)	0.31
**Masa consumption**					
No masa	591	956 (924, 989)	146	970 (875, 1074)	0.79
Masa	727	977 (951, 1003)	199	961 (918, 1005)	0.53
No masa + SUPP	146	1140 (1067, 1218)	34	1133 (1002, 1281)	0.94
Masa + SUPP	179	1186 (1119, 1257)	48	1239 (1096, 1401)	0.53
**Time spent in U.S**					
U.S Born	901	1015 (986, 1044)	231	985 (912, 1065)	0.48
Born outside U.S, been in U.S ≥15 years	293	1036 (987, 1088)	103	1072 (995, 1156)	0.45
Born outside U.S, been in U.S <15 years	389	973 (931, 1018)	73	1037 (956, 1124)	0.18
Missing	60	—	20	—	
**Primary language spoken at home**					
Primarily English	655	1021 (987, 1056)	160	965 (882, 1055)	0.24
Equal Spanish and English	337	995 (955, 1036)	95	1032 (938, 1135)	0.49
Primarily Spanish	644	1000 (961, 1040)	168	1041 (974, 1113)	0.31
Missing	7	—	4	—	

^a^ Abbreviations: enriched cereal grain product (ECGP), National Health and Nutrition Examination Survey (NHANES), red blood cell (RBC), ready-to-eat cereals (RTE), supplement use (SUPP); ^b^ Geometric means adjusted for age, education, poverty-income ratio (PIR), ethnicity, and body mass index (BMI); ^c^ Excluded those who did not attend the mobile examination center (2011–2016: N = 76, 2017–2018: N = 23) and those who had missing RBC folate concentrations (2011–2016: N = 91, 2017–2018: N = 20); *p*-values determined using Student’s *t*-test.

**Table 5:** Adjusted red blood cell (RBC) folate concentrations of U.S. Hispanic women of reproductive age (12–49 years), who were enriched cereal grain product (ECGP)^a^ only consumers from NHANES 2011–2018, stratified by acculturation factors and pre- and post-implementation of corn masa fortification.

	Time Period	n	Adjusted Geometric Mean ^b^ of RBC Folate, nmol/L (95% CI)	*p* Value (within Time Periods)	*p* Value (between Time Periods)	% with RBC Folate Concentration < 748 nmol/L ^c^ (95% CI)	*p* Value (within Time Periods)	*p* Value (between Time Periods)	Adjusted Odds Ratio ^d^ of Having an RBC Folate Concentration < 748 nmol/L (95% CI)
**Time spent in U.S**									
U.S Born	2011–2016	499	950 (918, 982)	REF		19.9 (16.2, 23.5)	REF		REF
Born outside U.S, been in U.S ≥ 15 years		164	975 (916, 1037)	0.47		20.3 (13.6, 27.0)	0.92		1.22 (0.70, 2.12)
Born outside U.S, been in U.S < 15 years		216	894 (844, 946)	0.07		27.7 (19.9, 35.5)	0.08		1.86 (1.00, 3.44)
U.S Born	2017–2018	143	918 (812, 1037)	REF	0.60	25.2 (18.0, 32.5)	REF	0.19	REF
Born outside U.S, been in U.S ≥15 years		59	1039 (929, 1162)	0.14	0.33	14.8 (5.4, 24.2)	0.09	0.35	0.40 (0.10, 1.50)
Born outside U.S, been in U.S <15 years		49	1018 (982, 1057)	0.11	<0.001	22.8 (13.7, 31.9)	0.69	0.42	0.39 (0.12, 1.25)
**Primary language spoken at home**									
Primarily English	2011–2016	365	936 (900, 972)	REF		21.2 (17.4, 25.1)	REF		REF
Equal Spanish and English		181	952 (893, 1015)	0.65		23.1 (16.6, 29.6)	0.62		1.17 (0.71, 1.92)
Primarily Spanish		361	941 (895, 990)	0.85		22.6 (17.6, 27.6)	0.66		1.35 (0.84, 2.17)
Primarily English	2017–2018	99	879 (776, 996)	REF	0.35	28.8 (16.6, 41.1)	REF	0.24	REF
Equal Spanish and English		59	958 (848, 1082)	0.34	0.93	25.3 (15.9, 34.6)	0.66	0.71	0.36 (0.06, 2.19)
Primarily Spanish		104	1034 (966, 1107)	0.03	0.03	15.8 (10.0, 21.5)	0.06	0.08	0.26 (0.06, 1.05)
**Masa consumption**									
No	2011–2016	435	946 (913, 981)	REF		22.1 (17.9, 26.3)	REF		REF
Yes		479	936 (909, 964)	0.65		21.7 (18.1, 25.4)	0.89		1.05 (0.71, 1.54)
No	2017–2018	124	942 (834, 1063)	REF	0.94	26.7 (18.2, 35.2)	REF	0.34	REF
Yes		141	953 (892, 1018)	0.87	0.64	19.4 (13.4, 25.3)	0.17	0.51	0.51 (0.12, 2.14)

^a^ Abbreviations: enriched cereal grain product (ECGP), National Health and Nutrition Examination Survey (NHANES), red blood cell (RBC); ^b^ Geometric means adjusted for age, education, poverty-income ratio (PIR), body mass index (BMI), and ethnicity; ^c^ Associated with NTD rate of ≥9 per 10,000 live births; ^d^ Odds ratios adjusted for age, education, PIR, BMI, and ethnicity; *p*-values determined using Student’s *t*-test. “Between time periods” examined differences pre- and post-fortification within the subpopulation; “Within time periods” examined differences between the subpopulation and reference group within the pre- or post-fortification time periods.

**Table 6:** Model-based estimation of neural tube defect (NTD) risk from red blood cell (RBC) folate distributions, with estimated NTD rates per 10,000 live births (uncertainty interval) post-implementation of corn masa fortification (NHANES^a^ 2017–2018).

	Estimated NTDs/10,000 Live Births (UI)	*p* Value
**All WRA**		
Non-Hispanic White	6.3 (4.6, 8.1)	
Hispanic	6.9 (5.1, 8.9)	
**WRA, ECGP-only**		
Non-Hispanic White	7.1 (5.3, 9.2)	REF
Hispanic	7.5 (5.6, 9.6)	0.37

^a^ Abbreviations: enriched cereal grain product (ECGP), National Health and Nutrition Examination Survey (NHANES), women of reproductive age (WRA); *p*-values from simulated estimation of population distributions.

## Data Availability

Publicly available datasets were analyzed in this study. The data can be found here: https://wwwn.cdc.gov/nchs/nhanes/default.aspx, accessed 20 August 2020.

## Supplementary Materials

Figure S1: Participant flowchart of NHANES 2011–2018 (https://www.mdpi.com/article/10.3390/nu13041325/s1).
