# Supplementary figures and images for: Impact of Voluntary Folic Acid Fortification of Corn Masa Flour on RBC Folate Concentrations in the U.S. (NHANES 2011–2018)
Source: Nutrients. 2021 Apr 16;13(4):1325. doi: 10.3390/nu13041325 (PMC8073626; doi:10.3390/nu13041325)

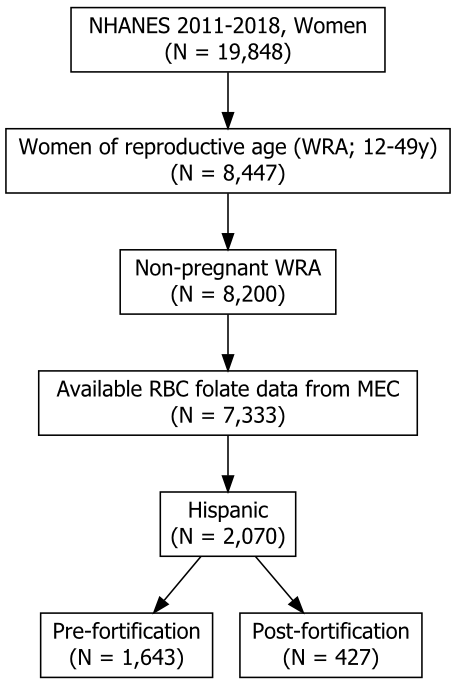

Supplement: Supplementary file 1 [file nutrients-13-01325-s001.zip › Figure_S1.tiff]
